# Review of the gastric physiology of disgust: Proto-nausea as an under-explored facet of the gut–brain axis

**DOI:** 10.1177/23982128241305890

**Published:** 2024-12-20

**Authors:** Sameer N. B. Alladin, Ruth Judson, Poppy Whittaker, Angela S. Attwood, Edwin S. Dalmaijer

**Affiliations:** School of Psychological Science, University of Bristol, Bristol, UK

**Keywords:** Disgust, electrogastrography, gastric rhythm, gut–brain axis, emotion

## Abstract

Humans feel visceral disgust when faced with potential contaminants like bodily effluvia. The emotion serves to reject potentially contaminated food and is paired with proto-nausea: alterations in gastric rhythm in response to disgust. Here, we offer a narrative synthesis of the existing literature on the effects of disgust on the stomach as measured through electrogastrography, a non-invasive technique that measures stomach activity with electrodes placed on the abdominal skin surface. After identifying and assessing 368 studies for eligibility and inclusion based on the Preferred Reporting Items for Systematic Reviews and Meta-Analyses process, we reviewed a final sample of only 10 articles that employed electrogastrography to assess gastric responses to unpleasant stimuli, including disgust elicitors. Reviewed findings illustrate that changes in gastric rhythm are associated with negatively valenced emotions, and most reliably with visceral disgust elicitors. This rhymes with recent evidence for a causal role of gastric state in reductions in visceral disgust avoidance. Because limitations in the reviewed body of work come from the low number of studies and relatively small sample sizes, we strongly encourage studies of proto-nausea in designs with higher statistical power, ideally paired with experimental manipulations of gastric state.

## Introduction

Disgust is recognised as one of the basic emotions that exist across cultures (Ekman and Friesen, 1971). At its core, disgust exists as a protective mechanism against the ingestion of potential toxins and contaminants (Angyal, 1941; Darwin, 1872) and is thus primarily inspired by bodily effluvia. In addition, disgust is thought to extend to a variety of additional illness-associated stimuli, including ectoparasites (Kupfer and Fessler, 2018), body envelope violations (Haidt et al., 1994), sexual emissions (De Jong et al., 2010; Freud, 1955 (1905)) and animals (Olatunji et al., 2008). Taxonomies of these separate stimuli and functions have been published elsewhere (Rozin and Fallon, 1987; Tybur et al., 2013). Here, we instead focus on *visceral disgust*, also known as *core disgust*, which we argue is a close collaboration between brain and body to avoid potential contaminants.

Even early conceptualisations of disgust are strikingly visceral, with Darwin (1872) emphasising the primacy of taste in the experience of disgust. He saw disgust as ‘a sensation rather more distinct in its nature, and refer[ring] to something revolting primarily in relation to the sense of taste, as actually perceived or vividly imagined’ (Darwin, 1872) and went on to indicate that disgust may also be brought on secondarily by ‘anything which causes a similar feeling, through the sense of smell, touch, and even of eyesight’ (Darwin, 1872). Particularly relevant is Darwin’s observation of disgusting stimuli eliciting a specific oral physiological response: contracted nostrils to restrict airflow and the activation of particular facial muscles that facilitate ‘[letting] an offensive morsel drop out’. One of the earliest modern accounts of disgust echoes this oral physiological focus, describing the emotion as ‘a specific reaction towards the waste products of the human and animal body’ aimed at avoiding their ingestion (Angyal, 1941).

The above casts disgust as a response to physical stimuli, while extensions into other domains (e.g. moral disgust) reflect ‘disgust’ as a metaphor (Danovitch and Bloom, 2009), lexical fallacy (Armstrong et al., 2021; Fiske, 2020) or mixture of emotions (Van der Eijk and Columbus, 2023). This echoes the Oral Origins hypothesis, which states that disgust evolved as a mechanism to orally reject substances that can cause harm and disease (Angyal, 1941; Rozin and Fallon, 1987). Others have since pointed out disgust is also evoked by non-oral disease vectors (Curtis et al., 2004) and emerges later in life than one might expect for evolutionary beneficial behaviour (Rottman, 2014; Stevenson et al., 2010; although see Dalmaijer and Armstrong, 2020). However, popular theoretical frameworks cast disease avoidance as disgust’s original purpose, with higher-order disgust developing as later extension that co-opted some of the existing functions (Rozin and Fallon, 1987; Stevenson et al., 2010; Tybur et al., 2013). With this review, we do not intend to solve the debate on disgust’s evolutionary origins and structure, but merely to explore the evidence for the orogastric nature of visceral disgust.

### Facial responses to disgust

Research on facial expressions of emotion is notoriously difficult, and the basic emotions of anger, disgust, fear, happiness, sadness and surprise (Ekman and Friesen, 1971) are not as reliable or specific as initially thought (Feldman Barrett et al., 2019). Despite this, it is compelling that disgust-related activity of facial muscles is parsimonious with the oral origins hypothesis. As Rozin and Fallon (1987) pointed out, the facial expression associated with disgust mostly features those parts involved in food consumption. That is, the contraction of the nostrils serves to restrict offensive odours, and the gaping of the mouth allows for contents to dribble out (Ekman and Friesen, 1971; Izard, 1971; Rozin et al., 1994). Several studies have indeed found involvement of the mouth in response to disgusting stimuli (De Jong et al., 2011; Olatunji et al., 2008; Shenhav and Mendes, 2014; Van Overveld et al., 2009), specifically finding that the levator labii (muscle involved in elevating the upper lip) and the corrugator (involved in frowning) show increased activation in response to (core) disgust elicitors.

The salivary glands are also part of the facial disgust response, with increased digastricus activity (muscle involved in saliva production) and saliva production (De Jong et al., 2011; Van Overveld et al., 2009). For instance, Van Overveld et al. (2009) found that disgusting stimuli elicited increased levator labii activity and increased saliva production. There was also an increased self-reported tendency to vomit, and this tendency to vomit was stronger for core disgust stimuli than blood-injury stimuli.

De Jong et al. (2011) found that physiological reactivity to core disgust in the form of saliva production and facial muscle activity was not significantly correlated with the subjective experience of disgust. This could suggest that physiological and self-reported responses to disgust are the outcomes of separate processes, potentially operating along separate neural pathways. It is also unclear whether there is a single face–mouth–stomach pathway in disgust, that is whether De Jong et al.’s findings extend further down the gastrointestinal tract.

Unfortunately, research on the ‘gastric’ in the ‘orogastric’ nature of disgust is less numerous or well-known. This is despite the importance to nausea and its role in the avoidance of gastrointestinal illness to the function of disgust (Stevenson et al., 2010). Here, we summarise work that used the non-invasive technique electrogastrography (EGG) to gauge gastric responses to disgusting or otherwise unpleasant stimuli. Our aim is to explore patterns in stomach rhythms and their association with the emotion of disgust. We discuss their direction and causality, and offer suggestions for future research.

### Proto-nausea: an alteration of typical gastric rhythms

One of the most characteristic manifestations of a disgust response is that of nausea, indicating the involvement of not only the oral but also the gastrointestinal system (Rozin and Fallon, 1987). Nausea is an uncomfortable symptom, typically in the stomach, that serves to prevent further ingestion of toxins (Stern et al., 2011). In most cases, it is correlated with alterations in gastric rhythm (Babic and Browning, 2014). While nausea is neither necessary nor sufficient for an emotional experience to be labelled as ‘disgust’ (Ekman and Friesen, 1971), it can accompany particularly strong experiences of the emotion. In addition, episodes of disgust can produce what we describe as ‘*proto-nausea*’: a measurable alteration of gastric state/rhythm without necessarily consciously experiencing feelings of nausea.

Proto-nausea can be measured using EGG, a technique that measures the electric potential over the stomach to identify a characteristic rhythm of around 3 cycles per minute (cpm), or 0.05 Hz. EGG is non-invasive and uses cutaneous electrodes placed on the abdominal skin over the stomach to record myoelectrical activity produced by interstitial cells of Cajal in the pacemaker region of the stomach (Koch and Stern, 2003). To identify typical stomach function, continuous EGG signal is processed to identify signal power per frequency, for example, using the Fournier transform. Because the signal of interest is slow (1–10 cpm), longer measurement durations are required to accurately measure gastric power (to ensure enough cycles are recorded). For example, diagnostic tests of gastric function typically involve a fasted-state and a postprandial EGG test, each of which are recommended to be at least 30 min (Yin and Chen, 2013).

In general, the dominant frequency in human EGG is 3 cpm and it is most apparent immediately before and after eating, but unstable after extended periods of fasting (Levine, 2005). Power in the normogastric range even (briefly) increases under sham feeding, but not for vagotomised patients, suggesting anticipatory cephalic-vagal modulation of stomach rhythms (Stern et al., 1989). Periods of nausea are characterised by tachygastria during which the dominant frequency shifts towards 4 to 9 cpm (Geldof et al., 1986; Levine, 2005; Stern et al., 1985, 1987), but bradygastria has also been reported and there are considerably individual differences in the pattern of dysrhythmias (Koch, 2014). Due to its ability to detect deviations from the regular 3 cpm rhythm, EGG can be an objective measure of nausea (Koch, 2014).

In the literature reviewed below, studies have sought or reported attenuations of EGG magnitude at 3 cpm, or shifts in signal magnitude towards lower (bradygastric range, 1–2.5 cpm) or higher frequencies (tachygastric range, 3.7–10 cpm). Such shifts are typically much more subtle that EGG patterns during nausea, which is why we refer to them as ‘proto-nausea’. Proto-nausea can also be experimentally manipulated using pharmacological interventions. For example, when paired with an incentivised exposure procedure (Dalmaijer et al., 2021), the anti-emetic drug domperidone reduces disgust avoidance behaviour in humans, but does not alter self-reported disgust (Nord et al., 2021).

Studies have reported various indices (e.g. reduced normogastria or increased tachygastria) and manipulations (e.g. pharmacologically normalising gastric state) of gastric involvement in disgust. In our reading, there is a mostly implicit notion in the literature that disgust disrupts normal gastric functioning without a clear consensus on how. We suggest that the most likely gastric response to disgust would be a nausea-like pattern, albeit reduced in magnitude. This would thus appear as a relative reduction in normogastric amplitude, paired with a relative increase in the bradygasrtic or tachygastric range. For ease of reference, we proposed to name this theoretical construct ‘proto-nausea’.

## Literature review of gastric responses to emotion

Here, we summarise and review work published on disgust that uses EGG. We used the search phrase ‘(TI = (disgust*) OR TI = (unpleasant)) AND (ALL = (electrogastrograph*) OR ALL = (core) OR ALL = (gastric) OR ALL = (orogastric) OR ALL = (visceral) OR ALL = (myoelectric*))’ on 16 November 2022 to identify 345 relevant articles included in Web of Science.

Additional studies were identified from Google Scholar using the combined keywords ‘disgust, stomach, gastric, electrogastrograph’ and by following references within articles. In addition, we used Google Scholar alerts for the terms ‘disgust’ and ‘EGG’ to capture studies published after 16 November 2022. During the submission and review process, an additional two articles were published, one of which referenced by a reviewer. Both of these were also included (Alladin et al., 2024; Porciello et al., 2024). This generated a further 35 articles.

**Figure 1.**
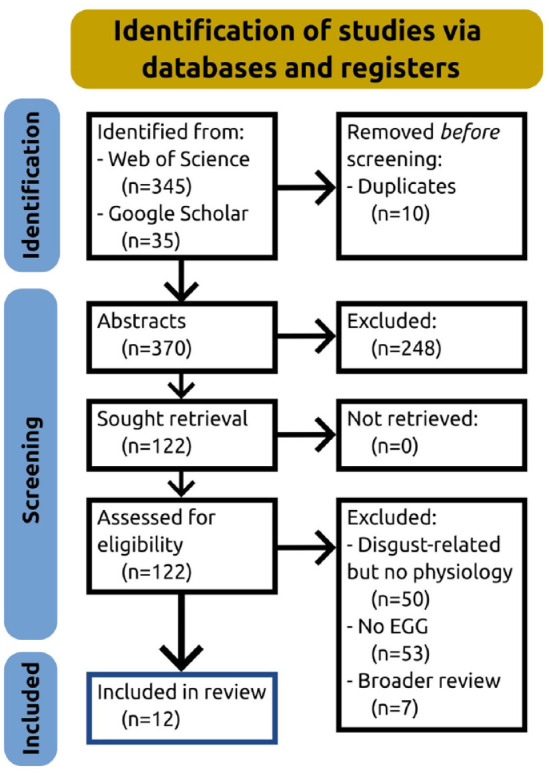
PRISMA flow diagram (Page et al., 2021) for the identification and inclusion of studies in this review.

Duplicates were removed, and records were screened by reviewing their abstracts. Remaining records were then selected or excluded by reading the full-text articles. This led to a final count of 12 studies that focussed on the use of EGG in emotion research, most of which including disgust, and one additional article that references EGG but does not use it (Nord et al., 2021). Details are shown in the Preferred Reporting Items for Systematic Reviews and Meta-Analyses (PRISMA) diagram in Figure 1.

Given the heterogeneity of study designs and outcome measures, as well as the relatively limited number of studies focused on the gastric nature of disgust, a narrative synthesis was pursued rather than a meta-analysis. This allows for linking together several studies, identifying points of convergence and suggesting future directions (Baumeister and Leary, 1997). We first summarise research that investigates gut involvement (particularly as measured with EGG) in emotional processes in general, before summarising research that focuses on disgust in particular. An overview of the reviewed studies is included in Table 1.

**Table 1.** Overview of the reviewed studies, including the type of stimuli used in each, and a short description of the findings relevant to disgust-induced proto-nausea.

Study	*N*	Channels	Stimuli	Proto-nausea effect
Baldaro et al. (2001)	40	1 (bipolar)	Gore disgust video	No gastric effect.
Stern et al. (2001)	38	1 (bipolar)	Appetising or disgusting food	Reduced normogastric power for disgusting stimuli.
Zhou et al. (2004)	32	1 (bipolar)	Unpleasant (including gore disgust) images	Reduced normogastric power for unpleasant stimuli.
Vianna and Tranel (2006)	16	1 (bipolar)	Negative valence emotionally salient (including disgust) videos	Reduced normogastric power for negatively valence stimuli; positive correlation between EGG amplitude and subjective arousal.
Vianna et al. (2009)	17	1 (bipolar)	Emotional imagery	Positive correlation between EGG amplitude and subjective arousal; negative correlation between EGG amplitude and vividness of imagery.
Harrison et al. (2010)	12	4 (bipolar)	Core and gore disgust videos	Increased tachygastric power for core (bodily effluvia) but not gore (body envelope violations) disgust.
Meissner et al. (2011)	26	1 (bipolar)	Core and gore disgust images	No spectral differences; positive correlation between bradygastric power and stimulus disgust ratings; positive correlation between bradygastric power and disgust sensitivity for stimuli.
Shenhav and Mendes (2014)	80^a^	1 (bipolar)	Core or gore disgust videos	Reduced normogastric amplitude for core (bodily effluvia) but not gore (body envelope violations) disgust. Negative correlation between normogastric power and disgust ratings for core disgust stimuli.
Valentina et al. (2018)	22	4 (common reference)	Positive and negative valence videos	Reduced normogastric power for positively and negatively valenced stimuli.
Tracy et al. (2019) ^c^	242^b^	N/A	Core and moral disgust images	Anti-emetic ginger had no statistically significant effect on core disgust ratings.
Nord & Dalmaijer et al. (2021) ^c^	25	N/A	Core disgust images	Anti-emetic domperidone reduced disgust avoidance.
Porciello et al. (2024)	31	1 (bipolar)	Core and gore disgust videos	No difference in normogastric or tachyhastric peak frequencies (amplitudes not reported). Negative relationship between stomach pH and disgust ratings (i.e. higher acidity related to higher disgust reports).
Alladin & Berry et al. (2024)	42	4 (common reference)	Core disgust images	No spectral differences between disgusting and neutral stimulus blocks in children aged 5–13 years.

a Divided into three groups of *N* = 28, *N* = 25 and *N* = 27 for between-participant comparisons.

b Divided into two groups of *N* = 121 each for between-participant comparisons.

c These studies did not record electrogastrography, but instead pharmacologically manipulated gastric state.

Where known, effective sample size (i.e. after exclusions) is reported. Channels refer to the recording, with *N* bipolar sensors producing *N*/2 channel, or *N* sensors with a common reference producing *N* channels.

Where possible, effect sizes have been calculated and included as part of section summaries. However, we advise caution when interpreting these, because the relatively small sample size in the reviewed studies would not have permitted detection of smaller effects.

### Gastric activity and emotion

Baldaro and colleagues (2001) were the first to explore the effect of highly aversive dynamic visual stimuli on respiratory and electrogastric activity in 40 female participants. They used two electrodes, one placed between the umbilicus and xiphoid process, and the other placed in the upper left quadrant. Using a sample of 40 female psychology students, they showed a surgery video and neutral video after 10 min of baseline recording. Despite self-report ratings indicating that the surgery video was significantly more unpleasant and more arousing that the neutral film, there was no effect on normogastric activity.

Zhou and colleagues (2004) explored the effect of viewing pleasant and unpleasant photographs on electrogastric activity. Their setup involved two electrodes, one placed to the left of the midline and the reference placed right of the midline. They divided 32 undergraduate students into two equal groups to view photographs from International Affective Picture System (IAPS) that were rated as being highly pleasant and calming, or as highly unpleasant and exciting. They found that unpleasant photographs reduced normogastric amplitude, while pleasant photographs had no effect. The reduction in normogastric activity compared with baseline for unpleasant stimuli was inconsistent with that of Baldaro et al. (2001). It is worth noting that the photographs used by Zhou et al. were essentially disgust-related, including bloody faces, mutilated limbs and dead bodies, which reflect the category of body envelope violations as a disgust elicitor. As such, their work could be considered to be disgust-related, though not explicitly stated as such.

Vianna and Tranel (2006) explored the effect of emotionally salient videos on gastric myoelectrical activity in a sample of 16 participants. Their setup comprised two Ag-AgCl electrodes and placed above the umbilicus and below the lower left rib. Their manipulation involved the use of 10 standardised videos that were deemed to elicit discrete emotions of happiness, disgust, fear and sadness, as well as a neutral video. They found evidence for gastric changes in response to emotional stimuli compared with neutral stimuli. Particularly, there was an increase in peak amplitude of the normogastric signal for happy emotions, but a decrease in activity for negatively valenced stimuli. There was also a strong positive correlation (*r* = 0.64, *p* = 0.018) between maximum amplitude and subjective rating of arousal level, indicating a meaningful relation between body processes and phenomenological experience of that emotion. However, the authors did not tease apart the effect for each negative emotion, particularly for the correlation between EGG peak power and self-reported emotional arousal. As such, it is unknown to what extent disgust stimuli contributed to this effect, particularly as their results show similar *z*-scores for disgust and fear stimuli, but substantially larger variability for sadness.

Vianna and colleagues (2009), rather than looking at the effects of viewing images or a video, employed an emotional imagery task. They explored the relations between arousal, valence and vividness and maximum spectral electrogastric values. Consistent with Vianna and Tranel (2006), there was a strong positive correlation between spectral values and subjective arousal ratings (*r* = 0.83, *p* < 0.01). They also found a strong negative relation between maximum spectral values and vividness (*r* = −0.75, *p* = 0.04), and no significant relation between vividness and arousal. To explain the latter finding, they suggest that vivid recall involves relying more on neural structures than on feelings within the body, thus producing lower amplitude signalling in the gastrointestinal tract. On the contrary, less vivid recall may involve a greater reliance on body signals as neural systems are not as heavily involved.

More recently, Valentina and colleagues (2018) conducted a study involving 22 healthy participants viewing 21 videos of positive, negative and neutral valences while EGG was recorded with a six-channel setup. They found evidence for both positive and negative stimuli decreasing normogastric peak power compared with neutral stimuli, but no differences between positive and negative stimuli. In addition, by comparing subjective emotional intensity (by grouping responses into low and high intensity), they found that low-intensity stimuli had a higher normogastric peak power than high-intensity stimuli. For the former finding (valence), they suggested that normogastric power may be modulated by the central nervous system inhibiting enteric nervous system activity, potentially associated with the fight-or-flight response. This, therefore, begs the question to what extent is bodily experience of emotion a top-down process – that is, is bodily experience inherently a bi-product of brain-based activity, or do body signals act on the brain in a bottom-up way that creates a feedback loop. The study of proto-nausea may be useful for addressing this question, particularly as we argue that proto-nausea reflects changes in gastric signal before or in the absence of conscious awareness.

In sum, while it appears that the electrogastrogram is responsive to emotion, results are mixed. Gastric signal was reported to increase (Vianna and Tranel, 2006), remain unchanged (Zhou et al., 2004) or decrease (Valentina et al., 2018) in response to positively valenced stimuli. More consistently reported was a general decrease in normogastric peak power for unpleasant emotional stimuli (Valentina et al., 2018; Vianna et al., 2009; Vianna and Tranel, 2006; Zhou et al., 2004). From the available statistical data provided by Zhou et al. (2004) and Vianna and Tranel (2006), these effects appear to be large (*d* = 0.78 in the work by Zhou et al., 2004, and η^2^ = 0.27 in the work by Vianna and Tranel, 2006).

### Gastric activity and visceral disgust

Stern and colleagues (2001) explored the effect of unappetizing (disgusting) food on gastric activity. This study involved 38 healthy participants, and an EGG setup that involved an electrode placed above the umbilicus, another placed diagonally upwards from this, and a reference placed to the right of the umbilicus. Participants were divided into two groups, one given appetising and the other unappetising foods. Participants in the unappetising condition rated their food as significantly more unpleasant, disgusting and less appetising compared with those in the appetising condition. Crucially, those in the unappetising group had a decrease in normogastric (3 cpm) power.

Research by Harrison et al. (2010) explored the between the brain and the gut in response to disgust. Twelve participants were shown videos depicting core disgust and body-boundary-violation (BBV) disgust stimuli as well as control videos. While viewing these videos, participants underwent cardiac measures, EGG and whole-brain functional magnetic resonance imaging (fMRI). In addition to these physiological measures, participants provided self-report ratings of how disgusted, faint and nauseated they felt from each video. The researchers found that both core and BBV disgust videos equally induced disgust, but core disgust elicited greater feelings of nausea than BBV. In terms of physiological responses, they found experienced core disgust to be related to tachygastria, a finding that is consistent with other work relating feelings of disgust and nausea to gastric dysrhythmias. BBV disgust, however, appeared to be more related to cardiac than gastric activity changes. It should be noted, however, that this research was conducted in a very small sample using reasonably short stimuli (2 min per condition) with control condition alternating with disgust conditions. As such, this may not have allowed sufficient timing and power due to a small sample to capture bradygastric and even normogastric changes.

Meissner and colleagues (2011) investigated gastric responses to disgusting pictures, and between gastric activity and disgust sensitivity and intensity. In a sample of 31 healthy participants, they administered 60 IAPS stimuli (30 highly disgusting and 30 with neutral content). Disgusting stimuli were further subdivided into highly arousing and moderately arousing (it should be noted that the moderately arousing stimuli consisted of a mix of core and body envelope violations, while the highly arousing stimuli consisted of mostly body envelope violations). Meissner et al. used a two-electrode setup, with both being placed on the skin above the abdomen. They observed no significant differences between the three categories for percentage of bradygastria, normogastria and tachygastria. However, they did find that bradygastric activity positively predicted disgust ratings for both highly arousing and moderately arousing pictures, and bradygastria also positively predicted disgust sensitivity for moderately arousing pictures. This finding is noteworthy given their suggestion to regard bradygastria as a ‘prodromal state of disgust-associated vomiting’ (p. 14), and more broadly as gastric dysrhythmias are particularly associated with nausea and vomiting (Xing, 2006). As such, it appears that the study of proto-nausea ought to include further exploration of the bradygastric band.

Shenhav and Mendes (2014) explored the effect of various videos on gastric reactivity (among other measures). Eighty participants divided into three groups (*N* = 28, *N* = 25 and *N* = 27) that were shown videos that displayed visceral/core disgust elicitors (pus, faeces and vomit), gore disgust (injuries) and neutral stimuli (e.g. scenes of animals in nature). Core and gore stimuli were chosen to avoid contamination across domains – that is, core disgust stimuli did not include bodily harm, and gore stimuli did not include bodily effluvia. Electrodes were placed similar to Stern et al. (2001), with the ground placed below the right rib. Participants rated core disgust and painful injury stimuli as more negative than control stimuli, with further analyses indicating that core disgust stimuli were rated as more disgusting than gore stimuli. Normogastric amplitude was significantly reduced in participants in the core disgust condition compared with other conditions. There was no difference between electrogastric response to gore disgust and neutral stimuli, suggesting that visceral stimuli had a unique effect on the stomach. Finally, there was a moderate negative correlation between subjective ratings of disgust and normogastric reactivity for visceral disgust stimuli (*r* = –0.47, *p* = 0.036), with higher disgust ratings being associated with more strongly reduced normogastric activity.

Porciello and colleagues (2024) combined EGG recordings with an ingestible sensor to measure gastric acidity. Male participants (*N* = 31) were shown 9-s videos in 24-video blocks for five different emotional categories: disgust, fear, sadness, happiness, and neutral. Videos in the disgust block contained both mutilation (‘gore’ disgust) and contamination scenarios (core disgust). EGG was measured with a one-channel bipolar montage (following Yin and Chen, 2013), and gastric acidity was measured using an ingestible sensor. Repeated-measures analysis of variance (ANOVAs) of normogastric and tachygastric peak frequencies showed main effects of emotional category, but no statistically significant post hoc differences. Interestingly, stomach acidity was negatively related to disgust ratings and positively to happiness ratings. It should be noted that differences in EGG amplitude were not reported, which makes it harder to compare these results with other EGG studies.

Alladin and colleagues (2024) studied disgust avoidance and proto-nausea in 5- to 13-year-old children (*N* = 45). Using a preferential looking task, they showed that children in this age bracket showed disgust avoidance: higher dwell times for neutral than core disgust (bodily effluvia) images. Using a four-sensor setup with a common reference, they recorded EGG during blocks in which neutral or core disgust (bodily effluvia) stimuli were shown. There was no difference in peak amplitude in the bradygastric, normogastric or tachygastric bands. The authors interpreted this as potential indication that children first learn to behave according to social norms around disgust (i.e. avoidance of bodily effluvia) and only later internalise this into an interoceptive response (proto-nausea), but they did note that gastric responses during stimulus blocks could have been diluted by the use of short cartoon insets to keep children’s attention on the task.

The reviewed studies present a varied picture on the effect of emotional stimuli on gastric rhythm. Amplitude in the normogastric range was reported to increase (Vianna and Tranel, 2006), decrease (Valentina et al., 2018) or be unaffected (Porciello et al., 2024; Zhou et al., 2004) by positively valenced stimuli, whereas non-disgust negatively valenced stimuli generally produced decreases (Valentina et al., 2018; Vianna and Tranel, 2006; Zhou et al., 2004), but not always (Baldaro et al., 2001; Porciello et al., 2024). For gore-related disgust, findings are inconsistent too, with some finding a decrease in normogastric EGG amplitude (Zhou et al., 2004) where others did not (Baldaro et al., 2001; Porciello et al., 2024; Shenhav and Mendes, 2014). More consistently reported are reductions in normogastric amplitude and/or bradygastria in response to visceral disgust elicitors (Meissner et al., 2011; Shenhav and Mendes, 2014; Stern et al., 2001), but not in children (Alladin et al., 2024).

In sum, there is evidence for an effect of visceral disgust on gastric activity, particularly in reduced normogastric amplitude. This appears to be a medium to large effect, with Cohen’s *d* ranging from 0.59 (Shenhav and Mendes, 2014, disgusting versus painful stimuli) to 0.77 (Stern et al., 2001, disgusting versus non-disgusting food). Alongside this, we see relations between tachygastria and experienced disgust (Harrison et al., 2010; Meissner et al., 2011), but methodological issues, particularly that of insufficient stimulus duration, may limit a fuller understanding of gastric responses to disgust. Overall, these findings offer support for the idea that visceral disgust is an orogastric phenomenon in the truest sense, as both oral and gastric organs are involved.

### Manipulating proto-nausea to study visceral disgust

The majority of existing work explores gastric responses to a variety of valenced stimuli. However, where previously discussed studies found that gastric activity was *associated* with disgust sensitivity, two studies suggest that this relationship could be *causal* by pharmacologically manipulating gastric rhythms to reduce disgust ratings or avoidance.

One such study used ginger (*Zingiber officinale*) supplements, which contain several ingredients with weak anti-emetic properties, including a competitive antagonist for 5-HT_3_ (serotonin) receptors with central and peripheral effects (Ernst and Pittler, 2000). A recent review of 15 meta-analyses on ginger’s anti-emetic efficacy concluded that the supplement is a safe treatment for nausea and vomiting (Li et al., 2024), but also notes limitations in the quality of existing research and mixed findings between different causes of nausea (e.g. sickness during pregnancy, post-operatively, or after chemotherapy).

In a series of experiments with a between-participants design, Tracy and colleagues (2019) explored the role of ginger in reducing feelings of disgust towards purity-offending stimuli (pictures of core disgust elicitors) and moral violation stimuli (vignettes). They found that self-reported disgust when viewing images of pathogen-disgust content did not decrease on ginger compared with a sugar placebo. They also found that ginger did not affect judgements related to non-purity-based moral domains, but it did reduce disgust for purity-based moral domains.

Tracy et al. (2019) suggested that the anti-emetic effect of ginger works for ‘real’ disgust emerging from a physiological response to pathogen-related moral stimuli (e.g. vignette about someone urinating in a pool), but not to non-pathogen-related moral stimuli (e.g. trolley dilemma). This is particularly interesting given that the serotonergic system has been found to modulate human moral systems (Siegel and Crockett, 2013). Furthermore, the serotonergic system has been linked to several functional reasons for vomiting, including toxic rejection (Rubio-Godoy et al., 2007).

Nord and colleagues (2021) conducted a pre-registered, randomised, double-blind, placebo-controlled, cross-over study of domperidone in 25 healthy participants. Domperidone is a peripheral dopamine antagonist that is commonly used as an anti-emetic drug to normalise gastric rhythm, but barely passes the blood–brain barrier (and indeed had no measurable central effects in a control experiment). Using an established eye-tracking paradigm that incentivised participants to directly look at disgusting stimuli (Dalmaijer et al., 2021), Nord and colleagues found a significant reduction in oculomotor disgust avoidance to bodily effluvia while participants were on domperidone. In sum, when proto-nausea was eliminated during several minutes of exposure to disgust elicitors, their avoidance was reduced. This suggests that the experience of disgust is directly related to gastric activity.

### Methodological and statistical issues

Studies included in this review typically used relatively small sample sizes with low statistical power, many of which employed between-participant designs that further reduced power. In addition, statistics were not always employed or interpreted in the most optimal way. Finally, there is inconsistency in methodological details specific to EGG such as the width of analysis windows and the number of sensors used for recordings.

Small samples are not per definition an obstacle to adequate statistical inferences. They can be used to detect known large effects, or in experiments with many trials per participant that help boost the power of appropriate statistical techniques (e.g. linear mixed-effects models). However, when used to investigate under-explored effects, studies with small samples can per definition only report null effects or large effect sizes. We thus caution that the current (relatively large) estimates of effect sizes are likely to turn out smaller if larger-scale replications are conducted.

A related issue is that some studies have reported null findings as real effects. For example, the effect of ginger (versus a sugar placebo) was originally reported as ‘marginally significant’ and treated as real, while test results were not statistically significant, *F*(1240) = 3.47, *p* = 0.064 (Tracy et al., 2019). Furthermore, corrections for multiple comparisons are not always appropriately implemented. This is relevant in the context of findings that rest on just-below threshold values, which would have been corrected to a null effect even through liberal approaches. One example of this is the difference of ratings to moderately disgusting stimuli between ginger and placebo, *t*(240) = 2.03, *p* = 0.04, which occurred in a family of 11 tests (Tracy et al., 2019). While these are promising results that deserve replication in a larger sample and a more powerful design, they are not as immediately compelling as their study’s abstracts suggest.

An issue specific to EGG is stimulus duration. Because gastric rhythms are so slow, long periods of data should be recorded to accurately capture them. A typical normogastric rhythm operates at 3 cpm and thus has a wavelength of 20 s. Bradygastric rhythms can be as low as 1 cpm, at a wavelength of 60 s. Hence, short stimulus durations will fail to accurately capture gastric signal magnitude. For example, Shenhav and Mendes (2014) noted that they lacked power to explore power in the bradygastric range despite using analysis windows that spanned 4 min of stimulus presentation.

In the existing literature, there is substantial variation in the number of electrodes used, with most studies employing two electrodes in a bipolar arrangement (one channel), some employing four with a common reference, and others employing eight to create four bipolar channels. We argue in favour of using more than one channel for two reasons: (1) increasing the likelihood of detecting the gastric signal and (2) collecting more data on the gastric signal. For the former, as there is substantial variation in the angular orientation of the stomach across individuals, using more electrodes offers more lead directions, as used by Mirizzi and Scafoglieri (1983) and Rebollo et al. (2018). This increases the likelihood of detecting the gastric signal and offers the possibility of choosing the best of multiple orientations. Second, using more than two electrodes facilitates collecting more data on the gastric signal across the stomach. This is especially important given that multiple regions of the stomach can produce or regenerate the slow wave signal, in addition to the dominant pacemaker situated in the corpus (Sanders et al., 2003). This may be particularly relevant for future work that wishes to explore the role of tachygastria in disgust, owing to the presence of a specific tachygastrial pacemaker in the distal stomach (Yin and Chen, 2013).

### Future directions

The most pressing limitations of the existing body of work on proto-nausea using EGG and disgust manipulations are summarised up in the previous section. Some are easily remedied by using larger sample sizes and within-participant designs where possible.

A problem more unique to EGG is that of its low frequencies of interest that require long measurement periods. Past research has employed shorter-than-ideal stimulus presentation durations. In addition to simply using longer trials, a solution could be to use traditional block designs so that signal magnitude in lower frequency bands can be more accurately computed for blocks of trials in the same condition. Addressing this methodological shortcoming is important for the study of proto-nausea, in particular given the association between bradygastria and the experiences of disgust and nausea (Meissner et al., 2011; Xing, 2006).

We suggested that the number and placement of sensors should be relatively numerous, to be sensitive to subtle fluctuations of the electrogastrogram over the stomach. Existing research typically approached data processing by picking the sensor with the highest signal-to-noise ratio or that looked the best (Levakov et al., 2023), which is incompatible with the suggestion to integrate signals across channels. Ideally, statistical tools should be developed that use the data from the entire stomach in a meaningful way. A promising example comes from Alladin et al. (2024), who employed a signal extraction pipeline that included Hampel filtering to reduce outlier effects, and an independent component analysis to exclude sources of non-gastric signals.

Finally, we see promising future avenues for the incorporation of EGG in visceral disgust research, particularly in designs that combine multiple physiological recordings and causal manipulations of gastric state (e.g. with domperidone or ginger). For instance, in the context of proto-nausea, it is worth exploring to how gut and brain interact in the experience of disgust by using neuroimaging alongside EGG. This can be challenging for tools like electroencephalography and magnetoencephalography, which measure neural signals of much higher frequencies (1–45 Hz or higher) than the typical electrogastrogram (<0.1 Hz). Similar approaches have been taken more broadly using fMRI, such as by Rebollo et al.’s (2018) work on resting-state gastric networks in the brain, and more recent work by Levakov et al. (2023) on brain-gastric phase synchronisation, and Mayeli et al.’s (2023) work on gut–brain mechanosensation. Applying a multi-technique approach within the context of disgust will, therefore, be especially useful, enabling direct comparisons between disgusting and neutral stimuli.

By integrating the electroencephalography (EEG) and EGG data with facial data (perhaps measured by facial electromyography, EMG, or facial landmark detection), research can elucidate causal directions for pathways involving the face, mouth, stomach, and brain. The use of pharmacological manipulations will be especially useful in this context, similar to the work of Nord et al. (2021) and Tracy et al. (2019). These studies offer evidence that normalising gastric rhythm reduces disgust, and future work should aim to elucidate the underlying neuro-gastric interactions that drive reported behavioural changes. Tangentially related, it would appear that exploring proto-nausea and causal manipulations of gastric state will also be useful to comparing and contrasting core disgust with other types of disgust, including moral disgust, which may be seen as a combination of disgust and anger (Armstrong et al., 2021).

### Conclusion

We reviewed the limited but promising literature on emotion and stomach rhythms as measured with EGG and described the concept of proto-nausea: changes in gastric rhythm in response to experiencing disgust. The reviewed findings illustrate that changes in gastric rhythm are associated with negatively valenced emotions, and most reliably with visceral disgust elicitors. Furthermore, there is causal evidence that changes in gastric state reduce visceral disgust avoidance. Several limitations were noted, particularly related to small sample sizes and methodological concerns regarding testing equipment. We see promising avenues for the incorporation of EGG in visceral disgust research, and especially in combination with neuroimaging techniques, facial EMG or landmark tracking, and eye-tracking to complement existing research. Such approaches will also benefit from causal manipulations of gastric state (e.g. with domperidone or ginger). Ultimately, disgust is an excellent model system for studying interoception and embodied emotions. It may well turn out to be, as Strohminger (2014) suggested, ‘the unlikely academic star of our time’.
